# Targeting Long Chain Acyl-CoA Synthetases for Cancer Therapy

**DOI:** 10.3390/ijms20153624

**Published:** 2019-07-24

**Authors:** Matteo Rossi Sebastiano, Georgia Konstantinidou

**Affiliations:** Institute of Pharmacology, University of Bern, 3010 Bern, Switzerland

**Keywords:** ACSL1, ACSL3, ACSL4, ACSL5, ACSL6, cancer, lipid metabolism, fatty acid, cancer therapy, therapy target

## Abstract

The deregulation of cancer cell metabolic networks is now recognized as one of the hallmarks of cancer. Abnormal lipid synthesis and extracellular lipid uptake are advantageous modifications fueling the needs of uncontrolled cancer cell proliferation. Fatty acids are placed at the crossroads of anabolic and catabolic pathways, as they are implicated in the synthesis of phospholipids and triacylglycerols, or they can undergo β-oxidation. Key players to these decisions are the long-chain acyl-CoA synthetases, which are enzymes that catalyze the activation of long-chain fatty acids of 12–22 carbons. Importantly, the long-chain acyl-CoA synthetases are deregulated in many types of tumors, providing a rationale for anti-tumor therapeutic opportunities. The purpose of this review is to summarize the last up-to-date findings regarding their role in cancer, and to discuss the related emerging tumor targeting opportunities.

## 1. Introduction

### 1.1. Fatty Acid Metabolism

Lipid metabolism is a wide and intertwined network that mediates multiple functions, such as energy production [1], thermoregulation [2] and the synthesis of signaling molecules [3], while studies have demonstrated that abnormal levels of lipids contribute to numerous pathologies such as cardiovascular diseases, metabolic diseases and cancer [4]. Fatty acid imbalances have a prognostic value, as their levels in serum can act as tumor biomarkers, or they can predict responses to therapy in multiple cancer types, including leukemia [5], non-small cell lung cancer [6,7], breast cancer [8] and colorectal cancer [9,10].

Alterations of main metabolic pathways [11], especially those involving glucose and lipids [12], are among the most striking metabolic features that span many different cancer types [13]. Moreover, it is now clear that different oncogenic drivers induce different metabolic phenotypes in cancer cells. Indeed, the lipid metabolic network remodeling can embrace multiple aspects; some tumors tend to increase the intake of extracellular fatty acids derived from the diet [14,15], while others tend to rely more on de novo fatty acid synthesis [1]. Most enzymes involved in this fatty acid synthesis are under the transcriptional control of the sterol regulatory element binding proteins (SREBPs). The serine/threonine kinase Akt (also known as protein kinase B (PKB)), through the mammalian target of rapamycin complex 1 (mTORC1), induces the expression of SREBP target genes in order to control de novo lipid synthesis [16]. In de novo lipid synthesis, the acetyl-CoA is the building block for fatty acids and can be generated from citrate or acetate. Citrate comes from either glycolysis followed by the tricarboxylic acid (TCA) cycle or from glutaminolysis followed by reductive carboxylation. Citrate is cleaved by ATP-citrate lyase (ACLY) to form acetyl-CoA and oxaloacetate. Next, acetyl-CoA is carboxylated by acetyl-CoA carboxylase (ACC) to form malonyl-CoA, and this latter produces palmitate in a reaction catalyzed by fatty acid synthase (FASN) [1]. Palmitate is a completely saturated fatty acid that is subjected to desaturation by desaturases. The stearoyl-CoA desaturase (SCD1), an oxygen-depend fatty-acyl desaturase, catalyzes the biosynthesis of Δ9 monounsaturated fatty acids (MUFA), oleic and palmitoleic acid, from stearic and palmitic acid. Interestingly, SCD1 inhibitors are shown to suppress the formation of tumors that rely on de novo fatty acid synthesis, and are more potent in suppressing cancer cell proliferation compared to ACC or FASN inhibitors [17,18]. However, recent evidence suggests that sapienate biosynthesis, through fatty acid desaturase 2 (FADS2), enables cancer cells to bypass the requirement for SCD1 [19]. Although these results suggest some new emerging targeting opportunities, they also predict a resistance to SCD1 inhibitors. On the other hand, some tumors, for instance those driven by oncogenic RAS, depend upon extracellularly-derived unsaturated fatty acids, and therefore, they are inherently resistant to SCD1 inhibition [15]. Indeed, the suppression of extracellularly-derived fatty acid activation is sufficient to trigger anti-tumor effects in KRAS-driven lung cancer [20]. Interestingly, fatty acid activation catalyzed by acyl-CoA synthetases (ACSs), is essential for the metabolism of both extracellularly-derived and de novo-synthesized fatty acids, suggesting that the development of pharmacological inhibitors against ACSs may trigger a potent anti-tumor response by overcoming a compensatory activation of one of the two pathways. Thus, the use ACSs inhibitors may be particularly relevant in cases of cancer cells that normally rely on de novo fatty acid synthesis, but in a hypoxic tumor environment, where the oxygen-dependent SCD1 is impaired, cells are forced to switch to extracellular fatty acid uptake for survival [15,21].

### 1.2. Acyl-CoA-Synthetases

Free fatty acids, depending on their chain length, can either freely pass the plasma membrane or be transported by specialized proteins [22,23], but in order to be channeled towards anabolic or catabolic pathways, they require a two-step activation with the following ATP-dependent reaction catalyzed by acyl-CoA synthetases (ACSs):Fatty Acid + ATP 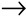 Acyl-AMP + PPi
Acyl-AMP + CoASH 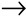 Acyl-CoA + AMP

ACSs, depending upon the fatty chain length of their preferred substrate, are divided into five enzyme families: Short-chain (C2–C4), medium-chain (C4–C12), long-chain (C12–C22), bubblegum (C14–C24) and very long-chain acyl-CoA (C18–C26) [3]. The synthesis of fatty acyl-CoAs is required for different physiological processes, among which: Proliferation and migration [24,25,26], energy fueling [20,27,28], steroid synthesis, reduction of pro-apoptotic free fatty acids [29] and glucose tolerance [30].

The acyl-CoA synthetase long-chain family (ACSLs) comprises five isoenzymes: ACSL1, ACSL3, ACSL4, ACSL5 and ACSL6 [31]. The five ACSL isoenzymes were identified, cloned and characterized by Yamamoto and his colleagues [32,33,34,35]. ACSLs catalyze substrates with a backbone of 12 to 22 carbons, but the isoenzymes differ in their substrate preference. For instance, ACSL1 has a marked preference for oleate and linoleate [36], ACSL3 for myristate, palmitate, arachidonate and eicosapentaenoate [3], ACSL4 for arachidonate [37], ACSL5 is reported to prefer palmitate, palmitoleate, oleate and linoleate [37] and ACSL6 has an equal preference for saturated and polyunsaturated fatty acids with a backbone of C16–C20 [38]. Furthermore, evidence supports the fact that their substrate preference is more complex, and is affected by many factors including membrane quality, coactivators, inhibitors, interactions with other enzymes, specific ACSL cellular location, specific ACSL tissue expression and posttranslational modifications [37,39].

Long-chain acyl-CoAs have multiple metabolic fates, including incorporation into ether lipids by fatty acyl-CoA reductases, incorporation into complex lipids and acylated proteins by acyl-CoA acyltransferases, convertion into acylcarnitines that enter the mitochondria for β-oxidation by carnitine palmitoyltransferase 1 (CPT1), or synthesis of bioactive lipids [40]. 

Moreover, an important aspect of ACSLs is their participation in lipid droplet biogenesis and the formation of triacylglycerols (TAG) and cholesteryl esters (CE), neutral lipids that constitute the cargo of lipid droplets [3,41,42]. This is in particular relevant in cancer because mounting evidence supports the fact that the number and size of lipid droplets are associated with cancer aggressiveness and resistance to therapy [43,44,45,46].

The ACSL isoforms differ also in their tissue and subcellular distribution. ACSL1 is localized in mitochondria, lipid droplets and microsomes of liver, heart, white and brown adipose tissue and skeletal muscle [3,47]. ACSL3 localizes to the periphery of the lipid droplets of lipogenic cells (for instance, hepatocytes) and thanks to its hydrophobic hairpin-like N-terminus, it is also present on the cytoplasmic face of the endoplasmatic reticulum (ER) [41,42,48]. It is controversial whether ACSL3 also plays a role in the trans-Golgi network, as it is implicated in the post-Golgi trafficking to the plasma membrane [49,50]. ACSL4 is reported to be present at the endosomes [51], peroxisomes [52], in the secretory pathway [49], at the plasma membrane [53] and at ER regions in contact with the mitochondria, named mitochondrial associated membranes [52]. The mechanism of ACSL4 membrane anchoring is not fully understood yet, but it has been proposed that specific splicing variants could express an analogous hydrophobic N-terminal region, as in the case for ACSL3 [52]. High levels of ACSL4 are mostly found in the adrenal gland, ovary, testis and brain [32]. ACSL5 is mainly located at the mitochondria, and it is highly expressed in intestinal mucosa, lung, liver, adrenal gland, kidney and brown adipose tissue [34,54]. ACSL6 is highly expressed in brain tissue [55], but its subcellular localization is still unclear. An extensive description about the subcellular localization and the specific function of ACSL isoforms can be found in the review by Tang and colleagues [4].

## 2. ACSLs in Cancer

The following chapter brings together the existing literature about the differential expression and, when available, the role of ACSLs in different types of cancers.

### 2.1. Colorectal Cancer

Three main ACSL isoforms have been found to be upregulated in colorectal cancer, ACSL1, ACSL4 and ACSL6 [28]. Importantly, high levels of *ACSL1* and/or *ACSL4* expression in patient tumor samples correlates with a worse prognosis [24,28,56]. Overexpression experiments performed in colorectal cancer cell lines evidenced that these isoforms mediate different aspects of carcinogenesis. ACSL1 overexpression elicits an upregulation of the epithelial to mesenchymal transition (EMT) markers N-cadherin and Slug, but is not sufficient for a complete EMT that is, in fact, only achieved by cells upon a combined overexpression of ACSL1, ACSL4 and SCD1 [56]. However, overexpression of either ACSL1 or ACSL4 in colorectal cancer cells is sufficient to increase wound healing, cell invasion [56] and proliferation [24] compared to empty vector control cells. Moreover, in colorectal cancer cells, ACSL4 overexpression and, to a lesser extent ACSL1, cause a shift of energy metabolism towards glucose utilization [24]. Conversely, the shRNA-mediated knockdown of ACSL1 or ACSL4 reduces cell proliferation in colorectal cancer cell lines, with a more marked effect elicited by the ACSL4 knockdown [24]. In support of these findings, another recent report evidenced that ACSL1 knockdown suppresses anchorage-independent growth and reduces cell migration [28]. 

Another interesting relationship between colorectal cancer and ACSLs involves the regulation of ACSL1 and ACSL4 by non-canonical micro-RNAs (miRNAs). Cruz-Gil and colleagues have shown that ACSL1, ACSL4 and SCD1 are all targets of miR-19b-1. miR-19b-1 is shown to reduce the neutral lipid content of colorectal cancer cells, and interestingly, its expression correlates with a better prognosis for stage II/III colorectal cancer patients. This suggests that miR-19b-1 can act as a prognostic marker in colorectal cancer [57]. 

Reports suggest different expression levels of *ACSL5* in human colorectal cancer. In the study of Gassler et al. [58] it is suggested that *ACSL5* expression and enzymatic activity is enhanced in adenomas and adenocarcinomas, while a more recent report from Hartmann et al. [59] describes a more complex picture. According to the latter study, *ACSL5* expression levels vary dramatically among colorectal cancer patients. Out of the 72 patient samples assessed by standard immunohistochemical staining, 41 were scored as low-ACSL5 while 31 as high-ACSL5. However, regardless of the differences in ACSL5 status, the survival rate of those patients was similar.

In a bioinformatic study in colorectal cancer using the Oncomine database, it was found that ACSL6 is upregulated compared to healthy tissue [28]. However, due to the lack of experimental evidence, its functional significance remains unknown.

### 2.2. Breast Cancer

Breast cancer shows a dysregulated lipid metabolism and alterations in fatty acid β-oxidation (FAO) levels. The main characteristic is the progressive increase of lipid droplets, a signature that is strongly associated with the transformation from normal tissue to invasive carcinoma [60]. Some breast cancer-relevant oncogenes have been reported to participate in this lipid network dysregulation in triple-negative breast cancer, on top of them Src [27] and Myc [61]. Moreover, in the context of hypoxia, lipid droplets have been shown to contribute to breast cancer cell survival upon hypoxia re-oxygenation. This is mediated by a hypoxia-inducible factor-1a (HIF-1a)-driven upregulation of the fatty acid binding protein 3 (FABP3) and FABP7 that are responsible for increased fatty acid uptake [46].

ACSL1 results to be transcriptionally upregulated in both estrogen receptor (ER)-negative [62], ER-positive [63] and HER2-positive [62] breast cancer subtypes, and its mRNA levels correlate with poor patient survival [28], but the specific mechanisms and functional significance of their upregulation are unclear.

ACSL3, although found to be upregulated in women with ER-negative breast cancer [64], has also been shown to be downregulated in triple-negative breast cancer [26]. In fact, in triple-negative breast cancer, ACSL3 interacts with the pro-metastatic protein CUB domain-containing protein 1 (CDCP1). CDCP1 inhibits the activity of ACSL3, resulting in a decreased fatty acid activation, and concomitantly, increased FAO. Indeed, in this context, high FAO/low lipid droplet abundance serve as a prognostic marker of triple-negative breast cancer metastatic potential [26]. 

ACSL4 is upregulated in quadruple-negative breast cancer (triple-negative breast cancer tumors that lack androgen receptor expression) [65]. In this setting, the expression level of *ACSL4* could be used as a diagnostic and prognostic biomarker. ACSL4 overexpression results in higher proliferation rates, higher invasion and anchorage independent growth in vitro and increased tumor burden in vivo [65]. Moreover, ACSL4 was shown to confer chemotherapy resistance in ER-positive breast cancer cell lines by upregulating the transcription of ATP-binding cassette transporters (ABC transporters), which are responsible for drug efflux [66]. 

*ACSL5* is upregulated in ER-negative, basal and normal-like subtypes. One of the interesting aspects of *ACSL5* expression is its association with estrogen and progesterone receptor expression, but the mechanism of this association remains elusive [62]. 

Taken together, this evidence highlights the complexity behind the expression of the different ACSL isoenzymes in breast cancer. 

### 2.3. Prostate Cancer

ACSL3 is expressed in both androgen-sensitive and castration-resistant prostate cancer, but in the latter its expression is dramatically higher [28,67]. Interestingly, ACSL3 overexpression leads to the upregulation of enzymes involved in steroids synthesis, one of the mechanisms underlying the development of castration-resistant prostate cancer [67]. In the castration-resistant prostate cancer context, ACSL3 promotes androgen synthesis through adrenal androgen dehydroepiandrosterone sulfate as a substrate, suggesting that ACSL3 may select for castration-resistant cancer cell populations. 

Of note, the current standard for the treatment of prostate cancer is androgen-deprivation treatment. However, after androgen-deprivation treatment, most patients develop castration-resistant prostate cancer after 2–3 years. Thus, it would be interesting to assess whether the increased *ACSL3* expression is concomitant to the cessation of the efficacy of the androgen-deprivation treatment and the development of castration-resistant prostate cancer. This would suggest that ACSL3-mediated steroidogenesis is an alternative way that cells utilize to cope with the androgen receptor signaling deficiency.

### 2.4. Melanoma

*ACSL3* is upregulated in melanoma, and its levels correlate with a worse patient prognosis, but the molecular mechanism regarding its role is unknown [28]. A signature of dysregulated lipid biosynthesis has been already observed by several reports [68]. Melanoma cells show high fatty acid synthase and acetyl-CoA carboxylase (key enzymes in the de novo pathway of fatty acid synthesis) compared to benign nevi, suggesting an increase in de novo fatty acid synthesis in melanoma patients [68]. High de novo fatty acid synthesis is accompanied by increased lipid droplet content, and altogether has been associated with increased therapy resistance and poor patient survival [69,70]. Because ACSL3 regulates lipid droplet biogenesis and maintenance [41], it would be interesting to investigate whether the lipid droplet increase and therapy resistance in melanoma patients is mediated by ACSL3.

### 2.5. Liver Cancer

Lipid metabolism occupies a central role in liver function. In liver, lipoproteins are assembled, and the regulation of the bodily distribution of various diet-derived lipid species has to pass through this physiologic and metabolic bottleneck. Hepatoma cell models show that ACSL3 is required for the assembly of very low density lipoproteins (VLDLs) [71] while ACSL1 spans a broader plethora of functions, among which are ER, peroxisome and mitochondrial lipid metabolism [72].

In a study of Nwosu et al. [73], an extensive analysis of publicly available patient-derived hepatocellular carcinoma expression data has been performed. The data were analyzed with a pathway finder algorithm, and the alteration of several pathways was assessed. Interestingly, lipid metabolism is altered in carcinoma samples, and among the ACSL family members, *ACSL3* and *ACSL4* expression results being up-regulated compared to a healthy liver. In parallel, *ACSL1* and *ACSL5* are down-regulated. These data are in agreement with the results of Chen et al. [28] and Liang et al. [74]. Finally, ACSL6 contributes to the accumulation of lipid droplets in fatty liver disease [75], with the latter being involved in the etiology of hepatocellular carcinoma [76]. Thus, it is tempting to speculate that the ACSL6-dependent lipid droplet increase could be required for the early stages of hepatocellular carcinoma development.

### 2.6. Lung Cancer

ACSL1 is reported to be downregulated in non-small cell lung cancer. Moreover, in vitro experiments show that RNA interference-mediated ACSL1 knockdown enhances the proliferation and invasiveness of non-small cell lung cancer cell lines [28]. These data indicate an anti-tumor role of ACSL1 in non-small cell lung cancer. On the other hand, bioinformatic analyses [28] and tumor tissue microarray (TMA) staining [20] showed that ACSL3 is up-regulated in lung cancer compared to the healthy lung tissue. Moreover, the H-score of ACSL3 (which takes into consideration the staining intensity in conjunction with the percentage of positive cells) appears overall reduced, with the ACSL3 levels being inversely proportional to the tumor stage. These results support a role of ACSL3 in tumor initiation, but not maintenance. This is additionally corroborated by a Kras-driven mouse model of lung tumorigenesis where the loss of Acsl3 suppressed the tumor onset [20]. Interestingly, in this context, oncogenic KRAS induces the ACSL3 promoter activity through the mammalian target of rapamycin complex 1 (mTORC1) regulation [20]. Whether this is a conserved mechanism in all KRAS-mutant tumors remains to be addressed. 

### 2.7. Soft Tissue Cancers

The expression levels of ACSLs in soft tissue cancers differ by isoenzyme and cancer type. Immunohistochemical staining revealed that ACSL3 is moderately to highly expressed in human fibrosarcomas, leiomyosarcomas and rhabdomyosarcomas [77]. On the other hand, low intensity staining was obtained for liposarcoma samples and a very scattered expression pattern was evidenced in dermatofibrosarcoma. Regarding ACSL4, a less defined pattern can be found; in fact, its expression is less homogeneous among the same tumor type. Notably, ACSL4 is highly expressed in some leiomyosarcomas and rhabdomyosarcomas, but is more consistently overexpressed in fibrosarcomas [77].

### 2.8. Blood Cancers

ACSL1 and ACSL6 are reported to be downregulated in a pan-leukemia dataset [28]. Accordingly, low levels of ACSL6 significantly correlate with poor patient survival in acute myelogenous leukemia, suggesting a tumor suppressing role in this context [28]. On the other hand, although it is known that lipid metabolism is dysregulated in acute myeloid leukemia [13], a small body of literature is available regarding the status and the role of ACSL3, ACSL4 and ACSL5 in blood cancers. Enhanced pathways are fatty acid synthesis [78] and FAO [78,79]. Therefore, it could be hypothesized that ACSL3, ACSL4 and ACSL5 are involved in the aforementioned metabolic re-shaping of cancer cells. 

## 3. Pharmacological Targeting of ACSLs

A few compounds are known for their ability to target ACSLs, but none are selective. The main issue about that is the conserved catalytic domain that makes the five ACSL isoenzymes have redundant catalytic functions [3]. The lead compound that is able to directly inhibit ACSL1, ACSL3 and ACSL4, by competing with fatty acids for their catalytic domain, is the fungal metabolite, Triacsin C [80]. At a higher dose, Triacsin C becomes also a competitive inhibitor of ACSL5 [81]. However, Triacsin C is toxic to cells at high concentrations and its polyunsaturated chain would make the pharmacokinetics and cell penetrance properties challenging.

Attempts to increase the activity of Triacsin C have been performed without a substantial success. In one study, the authors generated a library of six compounds by modifying the alkylidene terminal moiety of Triacsin C, and tested their activity in inhibiting the infective and replicative potential of Rotavirus using the lipid droplets content as a readout [82]. Interestingly, one compound named 1e showed a 50% lower median effective dose (ED_50_) than did Triacsin C. Another follow up study from Prior et al. [83] aimed to expand the previously synthetized library, and to test their inhibitory activity in a direct manner. Twelve different compounds were synthetized this time, and their capacity to inhibit the ACSL isoenzymes was tested either in the protein lysate or in living cells. Although some compounds showed a decrease in ^14^C-palmitate incorporation, none of the synthetized compounds showed any higher inhibition rate than Triacsin C. Another competitive ACSL inhibitor is 2-fluoropalmitic acid, a modified structure of palmitic acid with a higher affinity for ACSLs than the natural fatty acid [84]. The limitation of this drug is the high IC50. Moreover, no data are available regarding its affinity for any ACSL isoenzyme over others. Since the preference of ACSL3 and ACSL5 for palmitic acid is high, one could speculate that these may be the isoenzymes majorly inhibited by this molecule. Still more experiments have to be performed in order to understand which pharmacophore groups could confer specificity. In this way, the scientific community would finally be able to design more efficient and isoenzyme-selective analogs of Triacsin C.

Alternatively, an approach that could be used to interfere with ACSLs is the use of the antagonists of the peroxisome proliferator-activated receptors (PPARs), which transcriptionally regulate ACSLs [85,86]. Obviously, one drawback of this indirect inhibition is the high presence of off-target effects due to the various PPAR-controlled functions.

## 4. ACSLs as Therapeutic Targets in Cancer

**Figure 1:**
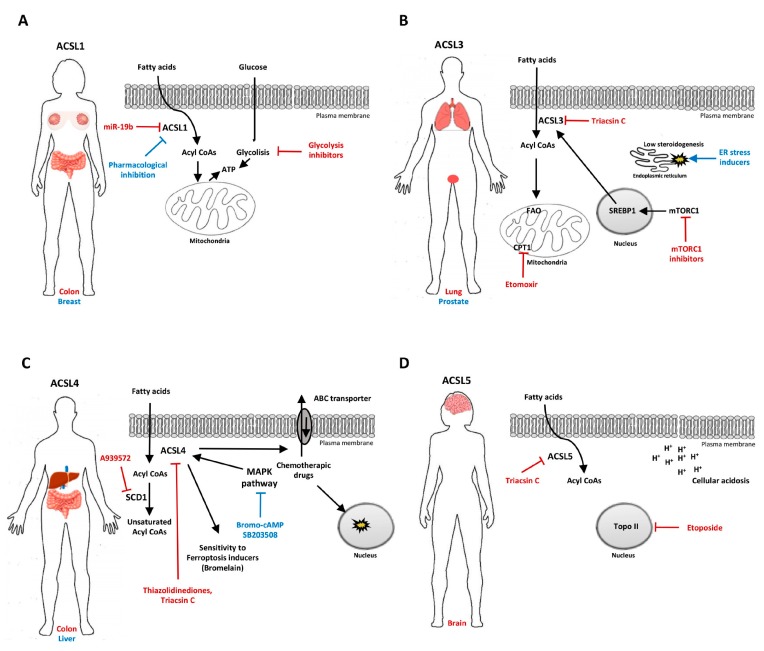
Proposed targeting approaches involving ACSL isoenzymes in cancer. (**A**) ACSL1 and glycolytic pathway co-targeting in colon cancer (red), and ACSL1 single targeting breast cancer (blue). (**B**) ACSL3, CPT1 and the mammalian target of rapamycin complex 1 (mTORC1) co-targeting in lung cancer (red) and the combination of ACSL3 inhibition with ER stress inducers in lung and prostate cancer. (**C**) Combined ACSL4 and SCD1 inhibition in colon cancer (red) and ACSL4 indirect targeting via the p38 MAPK pathway inhibition in liver cancer (blue). (**D**) ACSL5 and Topoisomerase II (Topo II) co-targeting with a potential therapeutic benefit in glioma.

Here, we report the cancer-specific effects of targeting individual ACSL isoenzymes (summarized in Table 1) and we discuss the emerging therapeutic opportunities (summarized in Figure 1).

### 4.1. ACSL1

The shRNA-mediated knockdown of ACSL1 in colorectal cancer shows reduced cell proliferation and migration [28]. This suggests a role of ACSL1 as a direct therapeutic target for colorectal cancer. Moreover, given the involvement of ACSL1 in favoring glycolysis [24], a potentially interesting treatment opportunity may arise from the combination of ACSL1 and glycolysis intermediates inhibition in colorectal cancer. 

As suggested by Cruz-Gill and colleagues [57], the inhibitory effect of miR-19b-1 on ACSL1, ACSL4 and SCD1 in colorectal carcinoma could be exploited as a therapeutic strategy. An in silico functional analysis performed in colorectal cancer cell lines revealed an effect of miR-19b-1 on focal adhesion formation and cytoskeleton remodeling which caused a reduction in cancer cell invasion and abolished their proliferation. This confirms the potential role of miR-19b-1 in ACSL1/4 axis targeting.

As previously stated, ACSL1 is upregulated in breast cancer, and its expression correlates with poor survival. RNAi experiments show that ACSL1 targeting reduces proliferation, colony formation and cell viability in breast cancer cell lines [28,62], thus rendering ACSL1 an attractive therapeutic target for breast, as well as colorectal cancer.

### 4.2. ACSL3

ACSL3 is found to be upregulated in fibrosarcomas, ER-negative, prostate cancer, melanoma, hepatocellular carcinomas and lung cancer, suggesting that targeting ACSL3 in this context may be clinically relevant for many cancers. Recent evidence in non-small cell lung cancer suggests that ACSL3 is important in maintaining the channeling of extracellularly-derived lipids to FAO [20]. Thus, FAO inhibitors such as Etomoxir, a carnitine palmitoyltransferase I (CPT1) inhibitor, could have an antitumor effect in lung cancer. On the other hand, in triple-negative breast cancer, ACSL3 is significantly reduced, resulting in high FAO/low lipid droplet abundance, and serves as a poor prognosis marker of the triple-negative breast cancer metastatic potential. However, also in this case, an inhibition of FAO could reduce cancer cell viability and suppress invasiveness.

We previously showed that KRAS drives the transcriptional upregulation of ACSL3 and ACSL4 in non-small cell lung cancer, and that this can be prevented by the inhibition of the mammalian target of rapamycin complex 1 (mTORC1) [20]. mTORC1 is a regulator of the sterol regulatory element-binding protein-1 (SREBP-1) prolipogenic transcription factor, which promotes aberrant proliferation in cancer cells [20]. Interestingly, both the ACSL3 and ACSL4, but not the ACSL1 and ACSL6 promoters, contain SREBP-binding sites. Moreover, mTORC1 inhibition abrogates the ACSL4-mediated chemotherapy resistance in breast cancer cells [66]. Thus, these results strongly support the idea that KRAS controls ACSL3 and ACSL4 through the mTORC1-SREBP signaling axis.

Some recent reports link ACSL3 and an unfolded protein response in cancer [91]. Thapsigargin-induced ER stress results in the downregulation of ACSL3, ACSL4 and solute carrier family 27 member 2 (SLC27A2) in lung cancer. Moreover, in a prostate cancer cell model, ACSL3 overexpression conferred resistance to tunicamycin-sensitive cancer cells [67]. These findings suggest that ACSL3 supports ER function to confer a survival advantage to cancer cells. ER stress triggers cancer cell death in many contexts [92,93,94,95], therefore ER stress inducers together with ACSL3 inhibition could result in enhanced antitumor response.

Finally, ACSL3 is able to drive steroidogenesis in castration-resistant prostatic cancer [67]. Thus, in this context, ACSL3 may be an attractive therapeutic target to prevent prostate cancer relapse.

### 4.3. ACSL4

Similar to ACSL1, ACSL4 is shown to have the potential for colorectal carcinoma treatment. Specific ACSL4 knockdown shows a reduced colorectal cancer cell proliferation [56]. On the other hand, the inhibition of ACSL4 with a non-toxic dose of Triacsin C does not have a significant impact on the cell viability of colorectal cancer cell lines. This is dramatically ameliorated by co-treatment with the SCD1 inhibitor, A939572 [56]. Accordingly, in vivo experiments performed in a xenograft model of colorectal carcinoma show that the treatment with Triacsin C is not effective in reducing the tumor burden. However, Triacsin C is sufficient to abolish the lipid droplet-mediated therapy resistance to the chemotherapeutic drugs, 5-fluorouracyl and oxaliplatin [90]. 

In breast cancer, another ACSL4-mediated resistance mechanism has been shown. The ABC transporters, ABCG2, ABCC4 and ABCC8 were identified as ACSL4-responsive genes involved in paclitaxel, cisplatin and doxorubicin resistance [66]. Indeed, combined ACSL4 inhibition with Triacsin C and treatment with the aforementioned chemotherapeutic drugs result in remarkable synergistic antitumor effects. In the light of these data, ACSL4 targeting could increase the efficacy of chemotherapy.

ACSL4 has been shown to be a ferroptosis-sensitizing factor [87]. In the study of Doll et al., the authors showed in mouse embryonic fibroblasts (MEFs) that ACSL4 mediates the incorporation of polyunsaturated fatty acids in phosphatidylethanolamine (PE) and phosphatidylinositol (PI). As a result, the shaping of the phospholipids landscape causes cells to become more sensitive to the ferroptotic inducers [96]. This occurs due to the intrinsic higher lipid peroxidation potential of lipids conjugated with unsaturated fatty acids [97,98]. Moreover, ACSL4 knockout MEFs are resistant to ferroptotic stimuli and the expression of ACSL4 or arachidonic acid supplementation is able to completely restore sensitivity [96]. Interestingly, the role of ACSL4 in sensitizing to ferroptosis has also been confirmed in basal-like breast cancer cell lines [96]. Additionally, in a study assessing the sensitivity of different colorectal carcinoma cell lines to Bromelain treatment (a mixture of proteolytic enzymes derived from pineapple stems), it was shown that ferroptosis was accompanied by higher levels of ACSL4 and lower levels of miRNAs targeting, among others, ACSL4 [25]. Intriguingly, the sensitivity to ferroptosis could be abrogated by RNAi-mediated ACSL4 knockdown or treatment with thiazolidinediones, a class of antidiabetic compounds, that among others, target ACSL4 and ameliorate the tissue demise in a murine model of ferroptosis. Altogether, these results suggest that ACSL4 inhibition is a viable therapeutic approach to prevent ferroptosis-related diseases. Interestingly, triggering ferroptosis could represent a therapeutic strategy for the treatment of many neoplasias, alone or in combination with chemoterapeutic agents [99]. Thus, it could be speculated that ACSL4 may represent a double-edged sword target, since both enhances cell proliferation and invasion and sensitizes cancer cells to ferroptotic stimuli.

The work of Liang and colleagues [74] shows that ACSL4 knockdown decreases the proliferation rates of hepatocellular carcinoma cell lines. Moreover, the expression of ACSL4 is driven by the p38 MAPK signaling pathway, and it can be inhibited by bromo-cAMP or SB203508 (a p38 MAPK inhibitor) treatment [74]. This suggests that ACSL4 may be indirectly targeted with p38 MAPK inhibitors.

### 4.4. ACSL5

*ACSL5* is upregulated in several cancers such as colorectal cancer, ER-negative, basal and normal-like breast cancer subtypes, suggesting that the identification of specific ACSL5 inhibitors is of paramount importance. Moreover, ACSL5 is frequently overexpressed in malignant gliomas [89]. Using gain- and loss-of-function experiments, Mashima et al. showed that ACSL5 selectively promotes human glioma cell survival under conditions of extracellular acidosis [88]. Moreover, Triacsin C enhances the antitumor efficacy of the chemotherapeutic drug etoposide [89]. These results suggest that ACSL5 may be critical for the malignant progression and metastatic dissemination of gliomas, and support the fact that targeting ACSL5 in this context may be an effective therapeutic strategy.

### 4.5. ACSL6

The data available for ACSL6 suggest that this isoenzyme has opposite roles in different cancers. In colorectal cancer it has been shown to be overexpressed, while in acute myelogenous, leukemia is downregulated, and is proposed to act as a tumor suppressor in this context. However, the lack of experimental evidence precludes further conclusions. Future investigations are needed in order to understand the role of this isoenzyme in different cancers.

## 5. Conclusions

Here we collected and summarized evidence that ACSLs are potential therapeutic targets in multiple cancer types (Figure 1 and Table 1). The individual ACSL isoenzymes, due to their different fatty acid chain length preference, tissue distribution and subcellular location, are able to channel specific fatty acids toward distinct metabolic fates. In cancer, the expression of most of the isoenzymes is amplified, and this feature can be used to drive rationalized therapeutic approaches. 

Validation of the whole-body mouse knockout of some isoenzymes such as ACSL3 [20], ACSL5 [100] and ACSL6 [101] does not result in lethality or any overt dysfunction which renders them cancer cell specific vulnerabilities. Nevertheless, still a great effort is needed towards developing the necessary medicinal chemistry in order to select adequate and selective inhibitors for this purpose.

There is significant caution in the field of metabolic targets progressing through clinical development due to extrinsic supply nullifying any effect of target inhibition [102]. For instance, it was shown that monoacylglycerol lipase (MAGL), by liberating free fatty acids from neutral lipid stores, controls the free fatty acid levels in cancer cells. Indeed, MAGL knockdown suppresses tumorigenesis in mice. However, the suppressed tumor growth that was observed after MAGL knockdown could be rescued by the high-fat diet in mice, indicating that exogenous sources of fatty acids can contribute to malignancy in cancers lacking MAGL activity. Since ACSLs are able to activate fatty acids deriving both from de novo and exogenous sources, a combined inhibition of MAGL and a tissue-relevant ACSL isoenzyme may result in a significant anti-tumor response. Further studies are warranted to assess combination therapies involving ACSL isoenzymes in cancer.

## Figures and Tables

**Table 1:** Impact of the deregulation of ACSL isoenzymes in cancer models.

Target Isoenzyme	Cancer Type	Effect	Reference
**ACSL1**	CRC cell line	**Overexpression:** enhanced wound healing, invasion, proliferation, glycolysis and EMT (this latter, only when combined with ACSL4 and stearoyl-CoA desaturase (SCD1) overexpression). **Knockdown:** decreased cell proliferation, migration, and anchorage-independent growth. **miR-19b-1 expression:** decreased invasion and proliferation.	[24,28,56,57]
	BC cell lines	**Knockdown:** decreased proliferation, colony formation and cell viability.	[28]
	NSCLC cell lines	**Knockdown:** enhanced proliferation and invasiveness of non-small cell lung cancer cell lines.	[28]
**ACSL3**	BC cell lines	**Knockdown:** decreased proliferation and viability, increased FAO/decreased lipid droplets content.	[26]
	PC cell lines	**Overexpression:** protection against ER stress inducers. Increased intracellular steroidogenesis.	[67]
	NSCLC cell lines	**Knockdown:** decreased cell proliferation, colony formation and reduced FAO.	[20]
	HCC	**Knockdown:** decreased very low density lipoprotein (VLDL) secretion.	[72]
	KRAS NSCLC GEM mouse model	**Total mouse knockout:** reduced tumor initiation/tumor burden.	[20]
**ACSL4**	CRC cell line	**Overexpression:** enhanced wound healing, invasion, proliferation and glycolysis. **Knockdown:** decreased cell proliferation and glycolysis, insensitivity to ferroptosis inducers. **miR-19b-1 expression:** decreased invasion and proliferation.	[24,56,57,87]
	HCC cell lines	**Knockdown:** reduced proliferation.	[74]
	ER+ BC cell lines	**Overexpression:** enhanced cell proliferation, increased drug efflux and chemotherapy resistance.	[65,66]
	Quadruple-negative BC cell lines	**Overexpression:** Increased invasion and anchorage independent growth in vitro and increased tumor burden in vivo.	[65,66]
**ACSL5**	Glioma cell lines	**Overexpression:** enhanced cell survival under extracellular acidosis. **Knockdown:** reduced cell survival under extracellular acidosis.	[88]
**ACSL6**	NA	NA	NA
**ACSL1, 3, 4, (5)**	ER+, ER− and BC cell lines	**Triacsin C:** decreased proliferation and decreased chemotherapy resistance.	[62,65]
	CRC cell lines	**Triacsin C:** enhanced sensitization to stearoyl-CoA desaturase (SCD1) inhibition treatment.	[56]
	Glioma xenograft mouse model	**Triacsin C:** sensitization to low dose etoposide treatment.	[89]
	CRC xenograft mouse model	**Triacsin C:** decreased lipid droplets content and increased sensitivity to chemotherapy.	[90]

## Abbreviations

HCC	Hepatocellular carcinoma
ER	Estrogen receptor
CRC	Colorectal Carcinoma
ACSL1	Acyl CoA long chain synthetase 1
ACSL3	Acyl CoA long chain synthetase 3
ACSL4	Acyl CoA long chain synthetase 4
ACSL5	Acyl CoA long chain synthetase 5
ACSL6	Acyl CoA long chain synthetase 6
EMT	Epithelial to Mesenchymal transition
SCD1	Stearoyl-CoA Desaturase 1
CRPC	Castration-resistant prostate cancer
BC	Breast cancer
FAO	Fatty Acid Oxidation
NSCLC	Non-small cell lung cancer
